# Medicine and Socialism

**Published:** 1890-02-15

**Authors:** 


### HYGIENE.

### MEDICINE AND SOCIALISM.

Dr. W. M. Polk, Professor of Obstetrics, Sc., in the University of New York, publishes in the *New York Medical Record*, Dec. 21st, a lecture on the "Relation of Medicine to the Problem of Socialism." Socialism, the author says, is the expression of the lower orders of their discontent with the conditions of life, and he traces back the movement nineteen hundred years to a village in Palestine. But the content of the people is dependent upon their physical well-being, the first essential being a healthy life. Yet as we pass from home to home, from tenement to tenement, we do not find the conditions of healthful life present, or even possible with the majority of the population. In conclusion, Dr. Polk says: "Let us shake off the exclusiveness of our teachings, and take the public more into confidence, speak directly to it of those laws of life and health which are each day becoming so necessary to it. Drive home to him whose property right is represented by his brain or by his muscle that his body is his capital, out of which his profit must come. Then go to Surplus, and show how to correct those evils which press so hard on the physical condition of those outside." Dr. Polk's paper is well worth attentive perusal; although we fear his aspirations are little less Utopian than those of Socialism.

